# Evaluation of artificial intelligence robot’s knowledge and reliability on dental implants and peri-implant phenotype

**DOI:** 10.1038/s41598-025-94576-z

**Published:** 2025-03-19

**Authors:** Mithat Terzi, Mustafa Cihan Yavuz, Tayyip Bicer, S. Kutalmış Buyuk

**Affiliations:** 1 Department of Periodontology, Faculty of Dentistry, Istanbul Medeniyet University, Istanbul, Turkey; 2 Department of Orthodontics, Faculty of Dentistry, Ordu University, Ordu, Turkey

**Keywords:** Artificial intelligence, Dental implant, Patient information, Peri-Implant, Dental implants, Peri-implantitis

## Abstract

The aim of this study was to evaluate the reliability and quality of information generated by ChatGPT regarding dental implants and peri-implant phenotypes. A structured questionnaire on these topics was presented to the AI-based chatbot, and its responses were assessed by dental professionals using a modified Global Quality Scale (GQS) and the DISCERN tool. The study included 60 participants divided into three professional groups: oral and maxillofacial surgeons, periodontologists, and general dental practitioners. While no statistically significant differences were observed among the groups (*p* > 0.05), oral and maxillofacial surgeons consistently assigned lower DISCERN and GQS scores compared to other professionals. The findings of this study suggest that ChatGPT has the potential to serve as a supplementary tool for patient information in dental implant procedures. However, its responses may lack the depth and specificity required for clinical decision-making. Dental professionals should exercise caution when relying on artificial intelligence (AI) -generated content and guide patients in interpreting such information. Future research should explore the variability of AI responses, assess multiple chatbot platforms, and investigate their integration into dental clinical practice.

## Introduction

Dental implants have revolutionized modern dentistry by integrating seamlessly with the maxilla-mandible and supporting peri-implant phenotypes. These phenotypes, which include factors such as keratinized mucosa width, mucosal thickness, and peri-implant bone characteristics, are critical for maintaining implant stability and overall oral health^1^. While the clinical success of dental implants has been well-documented, the integration of advanced technologies such as artificial intelligence (AI) is now reshaping this field by enhancing precision, efficiency, and predictive capabilities^2^.

In recent years, AI has gained traction in dental healthcare, particularly in dental implantology. Identifying optimal implant sites and enabling early detection of complications such as peri-implant bone loss, AI-driven tools have significantly enhanced treatment planning and procedural accuracy by analyzing radiographic data^3,4^These technologies also allow clinicians to create personalized treatment plans tailored to unique anatomical and clinical parameters, which improves patient outcomes in both routine and complex cases^5^.

Despite these advancements in dental implant technology, the role of AI in dental healthcare has received comparatively less attention^6^. While AI-driven tools and robotics have revolutionized dental procedures by enhancing precision and efficiency, particularly in dental implant placement and postoperative care, their reliability in specialized contexts such as peri-implant phenotypes remains underexplored^7,8^Moreover, AI’s ability to analyze large datasets and provide real-time recommendations has contributed to notable improvements in patient outcomes. However, further research is needed to fully understand the accuracy and dependability of AI-generated insights in these highly specialized areas^9^.

AI-powered chatbots like ChatGPT represent a new frontier in patient information and clinical support. These tools are designed to provide accessible and detailed information based on user queries, offering significant potential to enhance patient understanding of complex dental topics^10^. However, questions remain regarding their reliability and suitability for clinical decision-making. ChatGPT generates responses using probabilistic algorithms, which may result in variability and a lack of depth in the information provided^11^. To address these concerns, this study evaluates ChatGPT’s performance using validated assessment tools such as the DISCERN instrument and the Global Quality Scale (GQS), aiming to determine its potential as a reliable resource for patient information and clinical support in dental implant procedures.

Although this study focuses on ChatGPT, it is essential to position its findings within the broader context of AI chatbot platforms, including Google Bard and Microsoft Bing Chat. Comparative studies assessing the strengths and limitations of these platforms are critical to understanding their role in delivering accurate and dependable information for clinical and educational purposes^12^. The purpose of this study was to bridge the gap in the literature by examining the reliability, applicability, and broader implications of AI in dental implantology, laying a foundation for future advancements in this rapidly evolving field.

## Methods

The study design is cross-sectional and observational, focusing on participants’ evaluations of ChatGPT-generated responses. This design was chosen because it allows for a snapshot evaluation of the reliability and quality of AI-generated content without requiring longitudinal follow-up. The study was conducted online in February 2024, and participants were informed via email and social media platforms. All participants provided informed consent to participate in the study. The study was approved by Istanbul Hisar Hospital Intercontinental Clinical Research Ethics Committee (Report No: 24-21, Date: 12/13/2024). All methods were performed in accordance with relevant guidelines and regulations, including the principles outlined in the Declaration of Helsinki. Informed consent was obtained from all participants.

### Participant characteristics

The sample size was calculated using the G*Power program (version 3.1.9.7; Axel Buchner, University of Düsseldorf, Düsseldorf, Germany) to determine an effect size with 90% power, requiring a total sample size of 54 (effect size: 0.50). The study consisted of 60 individuals with 20 individuals in each group to enhance data reliability. The sample consists of 60 participants, divided into three groups: oral and maxillofacial surgeons, periodontologists, and general dental practitioners. Each group comprises 20 professionals. All providers are appropriately certified by relevant dental implant certifying institutions, ensuring their qualifications in dental implants and peri-implant phenotypes. They were tasked with evaluating the responses provided by ChatGPT to the same set of questions. A carefully designed questionnaire was created for participants to evaluate the responses provided by ChatGPT to the questions listed below:

- Can you provide information about dental implant?
- Can you provide information about peri-implant phenotype?

The questionnaire underwent a two-step validation process. First, it was reviewed by an expert panel of five dental professionals with significant academic and clinical expertise to ensure content validity. Second, a pilot test was conducted with 10 participants who did not take part in the main study. Feedback from the pilot test was incorporated to refine the questions and response evaluation criteria.

## Processes and evaluation procedures

The questions used in this study focused on dental implants and peri-implant phenotype. Separate chat windows were opened for each question to prevent previous answers from influencing subsequent responses. The responses provided by ChatGPT were recorded and sent to the participants for evaluation. Participants assessed the answers using the Global Quality Scale (GQS) and DISCERN tools. A double-blind methodology was employed during the study, to minimize bias and ensure the objectivity of evaluations. Participants evaluating the AI-generated responses were blinded to the specific aims of the study, ensuring that their assessments were not influenced by prior expectations or knowledge of the study’s objectives. Additionally, the evaluators tasked with scoring the responses using the DISCERN and GQS tools were blinded to the identity and professional background of other participants, as well as to each other’s scores. This approach was designed to prevent bias related to professional hierarchy or peer influence and to promote impartiality in the scoring process. The DISCERN and GQS scales were modified to better evaluate the responses provided by the AI chatbot.

The DISCERN tool was employed to evaluate the quality and reliability of health-related information disseminated on the Internet. This tool consists of 16 questions, divided into three sections assessing reliability, treatment options, and overall quality. To ensure objectivity and consistency in scoring, the following methodology was adopted:

- **Independent Scoring**: Two independent evaluators, both with significant expertise in dentistry, assessed each response provided by ChatGPT.
- **Training**: Prior to scoring, evaluators were trained using DISCERN guidelines, ensuring a consistent understanding of the evaluation criteria.
- **Double-Blind Process**: Evaluators were blinded to the source of the responses and to each other’s scores to eliminate potential bias.
- **Consensus Approach**: After individual evaluations, discrepancies between the two scores were resolved through discussion to reach a consensus.
- **Scoring Framework**: Each question within the DISCERN tool was rated on a scale of 1 to 5, following its standardized structure:
  - **Sect. 1**: Reliability of the source (questions 1–8).
  - **Sect. 2**: Treatment options (questions 9–15).
  - **Sect. 3**: Overall quality (question 16).

The total DISCERN score was categorized as follows:

- **15–26**: Very low quality.
- **27–38**: Poor quality.
- **39–50**: Average quality.
- **51–62**: Good quality.
- **63–75**: Excellent quality.

The GQS (Global Quality Scale) was used as a second tool to assess the quality and usefulness of the information provided to patients, consisting of five questions. Scores were calculated by summing the points for each section. Total GQS scores between 1 and 3 were classified as low to moderate quality, while scoring above 3 were classified as good to excellent quality. These tools allowed for a structured assessment of factors such as clarity, accuracy, and relevance of the information provided by ChatGPT.

### Statistical analysis

Statistical analyses were performed using SPSS software (SPSS Inc., Statistical Package for Social Sciences, version 20.0, Chicago, IL, USA). The normality of data distribution was assessed using the Shapiro- Wilk test and the non-parametric test were applied. The demographic characteristics were compared among groups using the Kruskal-Wallis test. The Mann-Whitney U-test was performed to compare GQS and DISCERN parameters among groups. Spearman’s correlation coefficients were calculated to evaluate potential correlations between DISCERN and GQS parameters. Statistical significance was set at *p* < 0.05.

## Results

Questions regarding dental implants and peri-implant phenotypes were prepared for Chat GPT-4.0, and its responses are presented in Table 1. When we asked the participants the question ‘How many dental implants do you apply per month?’, the answers were shown in Table 2. There was no statistically significant difference between the three groups (*p* > 0.05).

**Table 1:** Responses provided by chat GPT-4.0 to the questions asked.

Can you give me information about the dental implants?
1. Definition: A dental implant is a titanium post (like a tooth root) that is surgically positioned into the jawbone beneath the gum line. Once in place, it allows your dentist to mount replacement teeth or a bridge onto that area. 2. Components: - Implant: The titanium screw that serves as a root for the new teeth. This is the part that’s implanted into the jawbone. - Abutment: A connector that is placed on, or built into, the top of the dental implant, to which a dental crown will be attached. - Crown (or prosthetic tooth): This is the part of the tooth that you can see. It’s usually made of zirconium or porcelain for natural looks. 3. Advantages: - Durability: With proper care, dental implants can last many years, even a lifetime. - Appearance and Feel: They look and feel like natural teeth. - Oral health benefits: Unlike a tooth-supported bridge, implants don’t require reducing other teeth. They also prevent bone loss that occurs when teeth are missing. 4. Procedure: - Evaluation: Initial consultation, imaging, and jaw evaluation to determine feasibility. - Implant placement: Surgery to insert the titanium post into the jawbone. - Healing process: The bone around the implant heals in a process called osseointegration. This can take several months. - Abutment placement: Once healing is complete, an abutment is placed. - Crown placement: After the gum heals around the abutment, the artificial tooth (or teeth) is made and attached to the abutment. 5. Success Rate: The success rate of dental implants varies depending on the health of the individual and where in the jaw the implants are placed, but, in general, dental implants have a success rate of up to 98%. Proper care can ensure they last a lifetime. 6. Who can get dental implants? Generally, anyone healthy enough to undergo a routine dental extraction or oral surgery can be considered for an implant. They must have enough bone and healthy gums to hold the implant. Tobacco use, certain chronic diseases, or conditions like uncontrolled diabetes might decrease the success rate of dental implants. 7. Care: Like natural teeth, dental implants require regular check-ups and conscientious oral hygiene. 8. Cost: The cost can be quite high, depending on various factors like geographic location, the complexity of the procedure, and the number of implants needed. It’s worth noting that many dental insurance plans do not cover implants or only cover parts of the cost. 9. Potential Complications: Though dental implants typically have a high success rate, potential complications can include infection at the implant site, injury to surrounding structures, nerve damage, and sinus problems (for implants in the upper jaw). It’s important to consult with a dental professional to discuss the potential benefits, risks, and whether dental implants are the right option for you.
**Can you give me information about the peri-implant phenotype?**
The term “peri-implant phenotype” is used in the field of dentistry to describe the characteristics and qualities surrounding a dental implant. Dental implants are artificial tooth roots placed into the jaw to hold a replacement tooth or bridge. The peri-implant area includes the soft tissues (such as the gums) and hard tissues (like the jawbone) that are immediately adjacent to the implant. The phenotype of this area can influence the long-term success of a dental implant. Here are some key aspects of the peri-implant phenotype: Soft Tissue Phenotype 1. Keratinized Mucosa Width: Adequate width of keratinized mucosa around the implant is considered beneficial for maintaining peri-implant health. 2. Soft Tissue Biotype: Soft tissue biotypes can be categorized as “thin” or “thick.” Thick biotypes are generally more forgiving to surgical and prosthetic errors, while thin biotypes can be more prone to aesthetic complications and soft tissue recession. 3. Gingival Health: The health of the gingiva (gums) around the implant is vital. Inflammation and infection can lead to peri-implantitis, a potentially serious condition. Hard Tissue Phenotype 1. Bone Quality: The density and quality of the jawbone can affect the stability and osseointegration of the implant. 2.Bone Levels: Maintaining adequate bone levels around the implant is crucial for its long-term success. Bone loss can lead to implant failure. 3. Alveolar Ridge Architecture: The shape and size of the alveolar ridge (part of the jaw where the teeth sit) can affect implant placement and aesthetics. Functional Factors 1. Occlusal Load: Excessive forces or improper alignment can lead to complications such as implant component fracture or bone loss around the implant. 2. Parafunctional Habits: Habits like bruxism (tooth grinding) can negatively affect the implant and surrounding tissues. Aesthetic Factors 1. Soft Tissue Contour: The shape and contour of the soft tissue can impact the aesthetic outcome. 2. Implant Position: The position of the implant in relation to the bone and soft tissue can influence both function and aesthetics. Understanding the peri-implant phenotype can help dental professionals better plan treatment, predict outcomes, and manage complications. Monitoring these factors over time is essential for the long-term health and success of dental implants.

The DISCERN and GQS scores of ChatGPT 4.0’s responses regarding dental implants are presented in Table 3. Although no statistically significant difference was found between the groups, it was observed that oral and maxillofacial surgeons assigned lower scores across all sections of the DISCERN scale compared to general dental practitioners and periodontologists. According to the DISCERN scale, general dental practitioners provided the highest overall mean score of 46.95, followed by periodontologists with a total mean score of 43.65, and oral and maxillofacial surgeons with a total mean score of 43.60. Oral and maxillofacial surgeons assigned the lowest score (3.15) in the GQS evaluation.

The DISCERN and GQS scores of ChatGPT 4.0’s responses regarding peri-implant phenotype are presented in Table 4. No statistically significant differences were found among the groups. Oral and maxillofacial surgeons gave the lowest score on the DISCERN scale (45.05), while general dental practitioners assigned the lowest score on the GQS scale.

Kendall’s tau-b correlations between scores number of dental implants performed monthly, DISCERN and GQS parameters were shown in Table 5. When the scores were statistically evaluated, no significant difference was found (*p* > 0.05). However, it has been observed that as the number of dental implant applications performed in a month increases, the scores given decrease. 

**Table 2:** Number of dental implants performed monthly.

Number of Dental Implants	General Dental Practitioners	Periodontologists	Oral and Maxillofacial Surgeons	P*
0–50	18	13	15	0.761
51–100	1	2	1	
101–150	1	3	3	
151–200	0	1	0	
> 201	0	1	1	
	20	20	20	

*Results of Fisher’s Exact test.

**Table 3:** Comparison of DISCERN and GQS scores ChatGPT results for dental implant among groups.

Parameters		General Dentist (*n*=20)		Periodontologist (*n*=20)		Oral and Maxillofacial Surgeon (*n*=20)		P*
		Mean (SD)	Min.-Max.	Mean (SD)	Min.-Max.	Mean (SD)	Min.-Max.	
**DISCERN**	**Section 1**	23.85 (4.46)	13–32	23.40 (4.85)	8–29	23.30 (3.84)	18–32	0.703
	**Section 2**	23.10 (5.32)	12–34	20.25 (3.96)	7–26	20.30 (5.19)	14–35	0.161
	**Total Mean**	46.95 (8.24)	32–63	43.65 (8.17)	15–54	43.60 (8.74)	32–67	0.336
	**Section 3**	2.65 (0.93)	1–5	2.20 (0.70)	1–3	2.55 (1.10)	1–5	0.373
**GQS**		3.25 (1.07)	1–5	3.25 (0.91)	1–5	3.15 (0.93)	1–4	0.974

SD, standard deviation; Min, Minimum; Max, Maximum; *Results of Kruskal-Wallis test.

**Table 4:** Comparison of DISCERN and GQS scores ChatGPT results for Peri-implant phenotype among groups.

Parameters		General Dentists (*n* = 20)		Periodontologists (*n* = 20)		Oral and Maxillofacial Surgeons (*n* = 20)		*P*
		Mean (SD)	Min.-Max.	Mean (SD)	Min.-Max.	Mean (SD)	Min.-Max.	
**DISCERN**	**Section 1**	25.15 (4.97)	15–37	24.45 (4.74)	16–31	23.95 (6.05)	16–40	0.772^Δ^
	**Section 2**	23.50 (6.90)	9–35	22.25 (5.07)	16–34	21.10 (6.28)	14–35	0.243*
	**Total Mean**	48.65 (11.02)	26–71	46.70 (8.64)	33–64	45.05 (11.99)	30–75	0.567^Δ^
	**Section 3**	2.75 (0.85)	1–5	2.50 (1.00)	1–5	2.80 (1.11)	1–5	0.498*
**GQS**		3.20 (0.95)	2–5	3.45 (0.83)	2–5	3.30 (0.92)	1–4	0.642*

SD, standard deviation; Min, Minimum; Max, Maximum; ^Δ^ Results of one-way analysis of variance test; *Results of Kruskal-Wallis test.

## Discussion

This study evaluates the reliability and quality of information provided by ChatGPT in the context of dental implants and peri-implant phenotypes, contributing to the limited but growing body of research on AI in dentistry. The results highlight the potential of AI chatbots for patient information while underscoring the need for cautious implementation in dental clinical settings. This study provides insights into the utility and limitations of ChatGPT as an educational tool by comparing evaluations across professional groups. As AI continues to advance, its integration into clinical practice requires scrutiny, especially in terms of the accuracy and reliability of the information^13^. This study represents a crucial step in understanding how AI-powered chatbots like ChatGPT can be utilized in patient education and information within the dental implant domain, contributing to the growing body of knowledge in this area​.

In recent years, there have been very important developments in the field of AI. This rapidly increasing development trend in AI has also affected chatbots supported by AI, leading to the emergence of many AI chatbots. These chatbots interact with users and achieve results in line with the user’s wishes. Today, while it is used in many areas such as finance, education, and customer service, it has now taken its place in the field of health technology^13–16^. AI chatbots can always inform patients about different healthcare question with access to this information, it ensures that they are aware of their own health status and health practices. The most important point here is that the accuracy of the information provided is so significant^17,18^ Kurt Demirsoy et al.^19^ evaluated reliability of information about orthodontics produced by ChatGPT. ChatGPT responses were evaluated by three groups as orthodontics, fifth year dental student and individuals seeking orthodontic treatment. They should that ChatGPT had significant, potentially in terms of patient information about orthodontics. The finding of present study were consistent with recently published papers.

This study aimed to evaluate the information provided by the Chat-GPT 4.0 AI chatbot on dental implant and peri-implant phenotype topics. The analysis of Tables 2, 4 and 4, and 5 revealed no statistically significant differences among the groups (*p* > 0.05). The lack of significant differences can be attributed to several factors. There are no strict regulations limiting specialized procedures, such as dental implant placement, to specific specialties. This lack of restriction may result in similar clinical experiences across general dentists, periodontologists, and oral and maxillofacial surgeons, leading to comparable evaluations. All participants evaluated the same AI-generated content, which provided a standardized dataset. The uniformity of ChatGPT’s responses, unaffected by the users’ clinical expertise, likely contributed to the homogeneity in evaluations. A shared perception of the accuracy and reliability of AI-generated information among participants might have resulted in consistent critical assessments across groups, reducing variability. ChatGPT delivers neutral and generalized responses, reducing the influence of individual expertise on the evaluation process and leading to closer results across groups. Open-ended questions may elicit more nuanced and detailed responses, providing richer qualitative insights but potentially increasing variability and subjectivity in evaluations. In contrast, closed-ended questions offer standardized answers, which facilitate consistency and comparability but may limit the depth of information gathered. A mixed-format approach could balance these strengths, enabling both comprehensive understanding and reliable quantification.

The results indicate that ChatGPT’s responses, characterized by their consistency and accessibility, are best suited as a supplementary resource for patient information. While not a substitute for clinical decision-making, its ability to provide uniform and reliable information across diverse professional groups underscores its potential value in supporting patient understanding. An important consideration when evaluating AI chatbots is the potential variability in responses when the same question is prompted repeatedly. ChatGPT generates responses dynamically based on probabilistic algorithms. This variability could influence the perceived quality and reliability of the information provided, particularly when evaluated by different professionals. Such differences underscore the importance of including multiple AI platforms in future study designs to assess consistency across platforms and identify which systems offer the most reliable and reproducible information for clinical applications. Evaluating multiple platforms would also provide a more comprehensive understanding of how various AI tools perform under similar conditions, further guiding their integration into dental practice.

This study contributes significantly to the existing dental literature by exploring the reliability and quality of information provided by AI chatbots like ChatGPT in the context of dental implants and peri-implant phenotypes. While prior studies have evaluated the role of AI in dental treatment planning, surgical guidance, and patient follow-up, limited research exists on assessing the informational reliability of AI chatbots specifically tailored to dental applications. Additionally, the study sheds light on how different dental professional groups perceive AI-generated information, offering insights into its potential integration into clinical practice. Unlike traditional sources of patient education, AI chatbots provide instant, accessible, and standardized information, which is a novel advantage highlighted in this study. However, this research also emphasizes the need for cautious implementation of AI tools, given the varying perceptions and critical evaluations observed among dental professionals. This study encourages future comparative studies involving multiple AI platforms to further validate the findings and expand their applicability across diverse clinical settings.

An analysis of Table 5 showed a negative correlation between the number of dental implant applications performed in a month and the scores given by participants. This finding suggests that more experienced individuals may adopt a more critical approach to evaluating information. The modification of the DISCERN and GQS scales enhanced the ability to evaluate ChatGPT’s responses effectively. The optimized scales provided a more robust framework for assessing clarity, comprehensiveness, and neutrality, contributing to a better understanding of AI-generated content.

A key contribution of this study is its insight into the potential integration of AI-based systems into clinical practice, particularly for patient information on dental implant procedures. AI chatbots demonstrated promise in improving patient access to information and facilitating a better understanding of treatment options. Nevertheless, caution is required when implementing such systems in clinical workflows to ensure the accuracy and reliability of the information provided.

The results of this study demonstrate that ChatGPT can provide reliable and valuable information for patient information regarding dental implants and peri-implant phenotypes. However, it is important to acknowledge that other AI chatbot platforms may exhibit differing capabilities depending on the context. Comparative studies involving multiple platforms could offer a more comprehensive understanding of their relative advantages and limitations in dental practice.

### Limitations

This study has several limitations. The relatively small sample size of 60 participants may restrict the generalizability of the findings. Additionally, the study focused on three categories of dental professionals: General dental practitioners, periodontologists, and oral and maxillofacial surgeons excluding other relevant specialties such as prosthodontists. Including such specialists could have provided broader insights into AI-generated content related to dental implants and peri-implant phenotypes. Future research should incorporate a wider range of dental specialties and larger, more diverse participant groups to enhance the validity and applicability of the findings. Furthermore, the scope of the questionnaire was limited; a more comprehensive set of questions is needed to thoroughly evaluate the AI chatbot’s performance in dentistry. Furthermore, participants’ familiarity with AI technologies may have influenced their evaluations, with more experienced individuals potentially being more critical. Lastly, the assigned scores were influenced by the personal experiences and knowledge levels of the evaluators. This study is limited to the evaluation of ChatGPT as a single AI chatbot platform. Other platforms may offer varying levels of accuracy, reliability, and contextual relevance. Future research should include a comparative analysis of multiple AI platforms to provide a broader perspective on their applications in clinical settings.

## Conclusions

AI chatbots show great promise in providing general patient information related to dental implant procedures and peri-implant phenotype. However, it is important to emphasize that this information should not be considered as a substitute for academic or clinical expertise but rather as a supplementary tool for patient education. No statistically significant differences were observed between the scores given by dental professional groups, indicating a consistent perception of AI-generated content among clinicians with varying specialties.

**Table 5:** Kendall’s tau-b correlation coefficients between scores number of dental implants performed monthly, DISCERN and GQS parameters.

		Dental Implant					Peri-implant Phenotype				
		**DISCERN Sect. 1**	**DISCERN Sect. 2**	**DISCERN** **Total Score**	**DISCERN Sect. 3**	**GQS**	**DISCERN Sect. 1**	**DISCERN Sect. 2**	**DISCERN** **Total Score**	**DISCERN Sect. 3**	**GQS**
Number of dental implants performed monthly	r	−0.099	−0.034	−0.090	−0.211	−0.069	−0.009	0.112	0.071	−0.070	0.076
	P	0.357	0.748	0.391	0.072	0.556	0.931	0.290	0.500	0.550	0.515

## Data Availability

The datasets underpinning the outcomes of this study can be obtained from the corresponding author, TB, upon a reasonable and substantiated request.
